# HPV Prevalence in Colombian Women with Cervical Cancer: Implications for Vaccination in a Developing Country

**DOI:** 10.1155/2009/653598

**Published:** 2009-12-20

**Authors:** Raúl Murillo, Mónica Molano, Gilberto Martínez, Juan-Carlos Mejía, Oscar Gamboa

**Affiliations:** ^1^Subdirección Investigaciones, Instituto Nacional de Cancerología de Colombia, Bogota, Colombia; ^2^Grupo Investigación en Biología del Cáncer, Instituto Nacional de Cancerología de Colombia, Colombia; ^3^Ginecólogo Oncólogo, Clínica del Country de Bogotá, Colombia; ^4^Patología, Instituto Nacional de Cancerología de Colombia, Colombia

## Abstract

Human Papillomavirus (HPV) vaccines have been considered potentially cost-effective for the reduction of cervical cancer burden in developing countries; their effectiveness in a public health setting continues to be researched. We conducted an HPV prevalence survey among Colombian women with invasive cancer. Paraffin-embedded biopsies were obtained from one high-risk and one low-middle-risk regions. GP5+/GP6+ L1 primers, RLB assays, and E7 type specific PCR were used for HPV-DNA detection. 217 cases were analyzed with 97.7% HPV detection rate. HPV-16/18 prevalence was 63.1%; HPV-18 had lower occurrence in the high-risk population (13.8% versus 9.6%) allowing for the participation of less common HPV types; HPV-45 was present mainly in women under 50 and age-specific HPV type prevalence revealed significant differences. Multiple high-risk infections appeared in 16.6% of cases and represent a chance of replacement. Age-specific HPV prevalence and multiple high-risk infections might influence vaccine impact. Both factors highlight the role of HPVs other than 16/18, which should be considered in cost-effectiveness analyses for potential vaccine impact.

## 1. Introduction

Cervical cancer continues to be the major cause of cancer mortality among women in developing countries [1]. Virtually all cases of cervical cancer are attributable to persistent Human Papillomavirus (HPV) infections, leading to the conclusion that HPV infection is a necessary cause of the disease [2].

The new HPV vaccines are designed to prevent HPV 16 and 18 infections which are the cause of about 70% of invasive cervical cancer cases worldwide. Thus, they are considered to be one of the most cost-effective interventions for cervical cancer control, particularly in developing countries where cytology-based screening programs have not been successful [3].

HPV vaccine efficacy is 93%–100% for reducing CIN/2-3 lesions associated with HPV 16 and 18 types [4, 5]; consequently, it has been estimated that they can reduce the burden of cervical cancer up to 70% worldwide [6]. Furthermore, several studies reveal that no major variation exists in specific HPV type prevalence among invasive cervical cancer in different regions around the world, indicating that the impact of HPV vaccines on cervical cancer incidence and mortality is expected to be similar across continents, with a potential reduction of 65% in South/Central America [6]. 

Although various cost-effectiveness analyses, including Latin American countries, have been conducted based on available HPV prevalence information, there are some concerns about the inclusion of HPV vaccines in public health programs. The factor with the greatest influence on HPV 16/18 vaccine cost-effectiveness is price per vaccinated girl [7]; another influential factor is vaccine effectiveness, and data from Latin America show that reduction of cervical cancer incidence could range widely (55%–69%) depending on HPV 16/18 prevalence [8].

Due to ethical restrictions, the burden of cancer related to specific HPV types should be estimated based on prevalence surveys. The most important surveys have been summarized in global meta-analyses providing relevant information for designing HPV vaccines as well as for assessing their impact in all regions of the world. Some limitations on meta-analysis and original studies are the differences in PCR technologies used to determine HPV infection and the analysis of multiple infections [9].

The abovementioned factors are crucial when considering second generation vaccines with broader coverage of HPV types and the potential benefits of cross-protection with current vaccines [9, 10]. In light of this, there is a need to more precisely examine the prevalence of different HPV types in Latin America, in order to more successfully predict potential cost-effectiveness of the introduction of current and future HPV vaccines. The aim of our study was to determine the prevalence of different HPV types in cervical cancer (squamous cell carcinoma and adenocaricnoma) in two Colombian cities with different risks for cervical cancer (incidence and mortality).

## 2. Methods

The study protocol was approved by the Ethics Committee of the National Cancer Institute of Colombia and each participating institution provided further approval.

### 2.1. Case Selection

The cases were retrieved from 18 clinical centers in two Colombian cities categorized as high risk (Barranquilla) and low-middle risk (Bogota) according to incidence and mortality from cervical cancer [11, 12]. The biggest clinical centers representing different sectors within the Colombian health system were invited to participate in both cities (private medical care, health insurance companies, and public hospitals). Formalin-fixed paraffin-embedded biopsies were provided in sequential order by pathologists from each institution, corresponding to all available invasive cervical cancer histological diagnosis for 2006-2007.

Clinical records were reviewed and patients with a history of HIV or any other immunodeficiency condition were excluded as well as patients who had undergone chemotherapy or radiotherapy prior to specimen collection.

Tissues from paraffin blocks were processed at the National Cancer Institute of Colombia and all cases were reviewed by an expert pathologist who verified histopathological diagnosis, assigned diagnostic groups, and graded according to standard histological criteria [13].

### 2.2. DNA Extraction

After histopathological review, ten 5–10/*μ*m sections of formalin-fixed paraffinembedded tissue were sliced from each block and placed in sterile 1.5 mL Ependorf tubes with 1 mL octane. The sections were obtained after deep cutting into the block at least twice, and microtome blade and histotechnician gloves were changed for each case.

Paraffin was removed with consecutive rounds of octane extraction followed by 100% ethanol washes. After high-speed centrifugation of the tissues, samples were added with 10 *μ*L acetone and the tubes dried at 55°C. The samples were incubated in digestion buffer (100 *μ*L of 200 *μ*g/mL Proteinase K) for 3 hours at 55°C and 12 additional hours at 37°C. Gentle centrifugation was carried out and the enzyme inactivated for 10 minutes at 95°C. 10 *μ*L of the sample were used for PCR analysis after additional gentle centrifugation.

To assess the quality of the target DNA, all samples were prescreened using a 209-base-pair amplifying *β*-globin PCR with BPCO3 and BPCO5 primer combination [14]. If a specimen was initially *β*-globin negative, new sections were obtained from the paraffin block and the whole process was repeated once.

### 2.3. HPV Detection

HPV-DNA detection was performed by a standard GP5+/GP6+ PCR based assay, which allows for the detection of a broad spectrum of genital HPV types [14]. Subsequently, HPV-positive samples were subjected to EIA-HPV group-specific analysis using cocktail probes for high-risk and low-risk HPV [15]. The high-risk HPV cocktail contained oligoprobes for HPV 16, 18, 31, 33, 35, 39, 45, 51, 52, 56, 58, 59, 66, and 68 and the low-risk HPV contained oligoprobes for HPV 6, 11, 26, 34, 40, 42, 43, 44, 53, 54, 55, 57, 61, 70, 71, 72, 73, 81, 82, 83, 84, IS39, and CP6108 [16].

### 2.4. HPV Type Specific Detection

Detection of 37 individual HPV types was achieved using a Reverse Line Blot assay (RLB), with a previously described system [16]. Briefly, in this method 37 oligonucleotide probes containing a 5′-amino group were covalently attached to a membrane in parallel lines using a miniblotter. After binding of the oligos, the membrane was removed from the miniblotter. For hybridization, 10 *μ*L of the PCR products were added to 150 *μ*L 2X SSPE/0.1% SDS, and the PCR products were denatured for 10 minutes at 99°C and cooled in ice. The membrane (with the oligonucleotide probes) was incubated for 5 minutes at room temperature in 2X SSPE/0.1% SDS and placed in a miniblotter, in a way that the slots were perpendicular to the line pattern of the applied oligonucleotides. The slots were filled with the diluted PCR products (160 *μ*L) and hybridized for 60 minutes at appropriate temperature. The samples were removed by aspiration and the membrane was washed twice in 2X SSPE/0.5% SDS for 10 minutes at 51°C.

Subsequently the membrane was incubated with 1 : 4000 diluted peroxidase labelled streptavidin conjugate in 2X SSPE/0.5% SDS (Roche, Mannheim, Germany) for 1 hour and washed in 2X SSPE/0.5%. For chemiluminescent detection of hybridized DNA, the membrane was incubated for 2 minutes in 20 mL of ECL detection liquid (Amersham, Buckinghamshire, England) and exposed to a film (Hyperfilm; Amersham, Buckinghamshire, England) for 30 minutes. Films were then developed. For repeated use, the membranes were stripped and stored at 4°C.

### 2.5. HPV E7 Type-Specific PCR

HPV E7 type-specific PCR for 14 high-risk HPV types (HR-HPV) was used to analyze *β*-globin positive and HPV negative cases with the GP5+/GP6+ PCR-EIA. PCR primers and conditions were described previously [2, 17]. PCR product detection was performed under the same abovementioned conditions.

### 2.6. Statistics

Based on available data on incidence and number of cases for the two regions included in the study [11], independent sample size for each city was estimated and corrected for finite populations. A 5% precision and 57%/12.5% prevalence for 16/18 types, respectively [17], resulted in a sample size of 108 cases in Barranquilla and 140 in Bogota.

As described by Särndall et al. [18], prevalence as proportion of cases and the corresponding 95%CI were determined for each city (independent universes) as well as for the total number of cases. High-risk HPV (HR-HPV), low-risk HPV (LR-HPV) [19], and specific HPV type prevalence were determined for single and multiple infections. For additional estimates (histological types, cumulative prevalence, age-specific prevalence, impact of vaccination), high-risk multiple infections were assigned in proportional fractions to each genotype according to the distribution of their single infections.

The differences among continuous variables were analyzed with the Student's *t*-test and the Wilcoxon rank-sum test. The distribution of noncontinuous variables was analyzed with the chi-square test and Fisher's exact test.

## 3. Results

After reviewing inclusion criteria, biological specimens from 268 cases were collected in both cities. 20 cases were excluded (8 carcinoma in situ or less, 7 inadequate biological specimens, and 5 with no tumor or tissue from adjacent organs other than cervix uteri). 26 (10.5%) out of 248 cases were *β*-globin negative and DNA-HPV was not found in 5 cases (2%), leaving 217 cases for final analysis (94 from Barranquilla, 123 from Bogota).

The overall mean age was 51.5, with no significant variation between squamous cell carcinoma (53.2) and adenocarcinoma (51.3). Squamous cell carcinoma (SCC) corresponded to 86% and adenocarcinoma (ACC) to 11% of cases, with no significant differences between cities (Table 1).

Table 2 describes 24 HPV types identified in our study either in single or in multiple infections: 15 HR-HPV, 4 LR-HPV, 3 probable HR-HPV, and 2 undetermined risk types [19]. In the high-risk city (Barranquilla) 1.1% of cases were found with no HR-HPV types corresponding to 0.3% overall; for these cases single infections with low-risk types were identified (HPV-44 and other unspecified LR-HPV).

HPV-16 was the most common type (50.4% and 52.1% in Bogota and Barranquilla, resp.), followed by HPV-18 (13.8% and 9.6%), HPV-45 (9.7% and 6.4%), HPV-31 (7.3% and 4.2%), and HPV-58 (6.5% and 4.2%). For the low-middle-risk city (Bogota) the first 5 abovementioned HPV types were observed in 87.7% of cases, while in the high-risk city these types corresponded to only 65.9% of cases (*P* = .04), allowing for a higher participation of less common HPV types.

HPV types found in SCC had a similar distribution to other published results from around the globe since most of the cases corresponded to the SCC histologic category (over 80% in Table 1). The 5 most prevalent HPV types accounted for 82.3% of SCC; in contrast, only 3 HPV types (16, 18, and 31) accounted for 94.6% of ACC cases (Table 3). HPV types 18 and 31 were more prevalent among ACC (32.9% and 14.9%) than they were in SCC (10.8% and 4.5) (*P* > .05), and HPV-44 (the unique specific LR-HPV identified in a single infection) was observed only in SCC.

**Figure 1:**
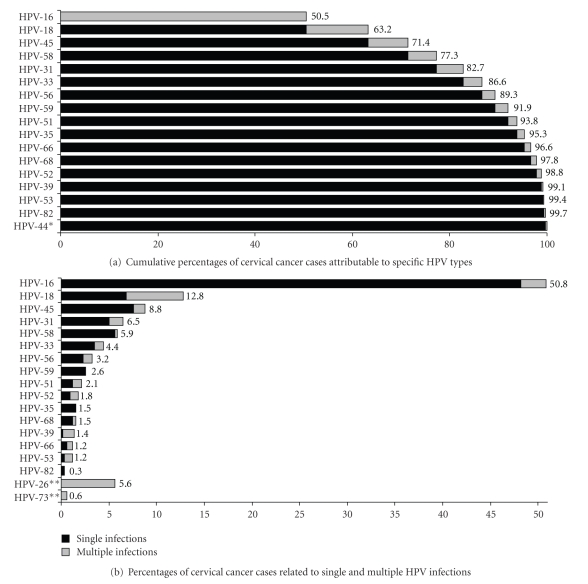
Cervical cancer cases and related HPV infections. (a) Only HPV types counted as single infections are included. Every cervical cancer case is attributable to solely one HPV type. Cases with multiple HR-HPV infections were proportionally distributed as described in the methods section. (b) Only HR-HPV types are included. HR-LR HPV coinfections (30.6% of multiple infections) were assumed as single infections for the carcinogenic process. *LR-HPV type with single infections as described in Table 2. **HR-HPV types observed exclusively in multiple infections.

Out of the total number of cases, 16.6% had multiple infections. Simultaneous occurrence of HPV types was present in 5% of HPV-16-associated cases, 47% of HPV-18, 14% of HPV-45, 23% of HPV-31, and 5% of HPV-58 (Figure 1). The majority of HPV-18 multiple infections were the combination with HPV-26 (Table 2). After assigning high-risk multiple infections in proportional fractions to each genotype according to the distribution of their single infections, 63.2% of cases were attributed to HPVs 16/18 (Figure 1).

**Figure 2:**
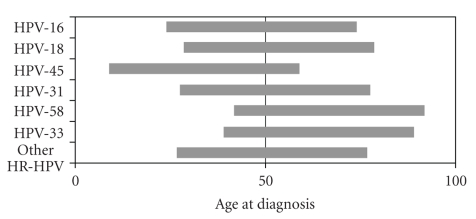
Distribution of HPV types over and under age 50. Each bar represents 100% of cases for each HPV type. HR-HPV types counted in less than 10 cases were grouped as other HR-HPV. Single infections assumed after distribution of multiple infections.

HPV types varied among women depending upon age at diagnosis of cervical cancer. For women under 50 the most common types were HPV-16 (55.4%), HPV-45 (14.3%), HPV-18 (10.1%), and HPV-31 (5.4%); for women 50-59, the most common types were HPV-16 (55.3%), HPV-18 (21.8%), HPV-58 (8%), and HPV-56 (3.4%); for women over 60, the most common types were HPV-16 (37.9%), HPV-58 (11.9%), HPV-33 (10.7%), and HPV-31 (9.8%). When considering age distribution for a given HPV type, HPVs 16 and 18 distributed evenly across age groups; most HPV-45 infections occurred before age 50 (82%, *P* = .02), and less common types (HPV 58 and 33) occurred mostly after age 50 and even over 60 (*P* > .05) (Figure 2).

## 4. Discussion

Our study is the largest on the subject in Colombia and one of the few published in Latin America. Previous information from Colombia included 125 cases in two studies with no ACC [20].

Several reports indicate that ACC has increased as a result of successful cervical cancer screening programs; however, the distribution of histological types in our report is similar to other studies in the region [20], and there were no differences among high-risk and low-middle-risk areas, regardless of differences in performance of cytology-based programs.

Our data show good sensitivity for the combined techniques of HPV detection, with better performance than in previous studies from Colombia, and similar to that from other regional reports and from developed nations [20, 21]. HPV-DNA detection was carried out using GP5+/GP6+ L1 primers, RLB assays, and E7 type specific PCR, allowing for 98% HPV detection among *β*-globin positive samples, and overall 99% HR-HPV detection among HPV positives. The high detection rates assure avoiding underestimation of HPV prevalence particularly for HR-HPV where all cases reported a specific type.

The largest previous Colombian survey included 87 cases with a restricted 72.4% HPV detection rate, in which only five HR-HPV types were analyzed using MY09/11 (exclusive of HPV-45 which played an important role in our study) [20]. A study that compared GP5+/GP6+ and MY09/11 showed similar global HPV detection sensitivity, but lower sensitivity detecting multiple infections with GP5+/GP6+ [22]; however, the differences between methods were not assessed and there were problems in primer design since the PCR product in the study did not include all regions corresponding to type-specific oligonucleotide probes for GP5+/GP6+.

In our study, type specific prevalence was similar to that found in aggregative analysis, where HPVs 16/18 are the most frequent [21]. As in those reports, our data likewise showed lower HPV 16/18 participation (63.1%) than observed in developed countries and in worldwide estimates [21]. Our results reproduce findings indicating that the role of HPV-18 in higher-risk areas might be slightly lower; consequently, the participation of rare HR-HPV becomes more relevant, such as in the case of Barranquilla (Table 2). Nevertheless, there were no significant differences between the two Colombian cities due to the lower prevalence found in Barranquilla.

As regards ADC, HPV-18 was significantly more prevalent than in SCC (34.3%) (Table 3), and these results were coincident with previous evidence [21]. However, less common HR-HPV types, such as HPV-31, were more prevalent in ADC in our study (particularly in the high risk city), and HPV-45 was not attributed to any ADC case after distribution of multiple infections.

When we turn our attention to the participation of multiple HPV types in a single case, we find out that it correlates with population risk and is coherent with the higher prevalence of multiple infections in developing countries: Africa 12.4%, South/Central America 11%, Asia 7.8%, Europe 6.2%, and North America 5.1% [20].

The existence of multiple infections increases the possibility of HPV replacement following vaccination and should be taken into account as a potential decliner of vaccine impact over time, mainly in developing nations where population risk and number of HPV types are higher. This issue is independent from that of assigning a specific HPV type to a specific lesion which is the main concern of vaccine efficacy in clinical trials [23].

Multiple infections have been dealt with differently in several studies: most estimates assume unique multiple-infection several times for different HPV types, resulting in overestimation of type specific prevalence [9, 20]. In other cases, multiple infections have been assigned in proportional fractions to each genotype but counted only once as in our study [17]. In order to ensure that specimen analysis met the highest international standards, we adhered to a strict protocol that greatly reduced the possibility of contamination. In our study, the percentage of multiple HR-HPV infections was higher than in reports from Africa (16.6% versus 12.4%) but occupied a mean position when compared to data from Latin American countries which ranges from 4.3% in Brazil to 33.3% in Mexico [20, 24]. Only 3 out of 8 regional reports show multiple infections under 10%.

Even so, existing information on multiple infections lacks consistency making it difficult to use for vaccine assessment: differences in PCR technique sensitivity are greater for detecting multiple infections, several reports do not present data on multiple infections, and studies reporting data on the topic use different structures. Thus, detailed information on multiple HR-HPV infections ought to be more relevant for developing countries, and this is particularly important for HPV-18. The 47% of multiple infections among HPV-18 in our study (Figure 1) is higher than the percentage in a worldwide metanalysis (20%) but similar to reports from Peru (56%) and lower than reports from Paraguay (90%) [17, 25, 26]. However, most HPV-18 multiple infections in our study occurred with a probable high-risk type (HPV-26) indicating a low chance of replacement.

In Colombia, as in other Latin American countries, a double peak of HPV prevalence and incidence among women with normal cytology has been described: after an initial high HR-HPV incidence in young women, there is a decline that goes up again around menopause [21]. In our report, type-specific HPV prevalence among women under age 50 was different than prevalence among postmenopausal women with invasive cancer; likewise, prevalence after ten years since the second incidence peak was also different (prevalence over age 60). 

Differential age-specific type prevalence might have different effects on global vaccine impact according to age-specific cervical cancer incidence. Thus, in Colombia less than 50% of invasive cancer occurs under age 50 which would imply a slightly lower impact of HPV 16/18 vaccines when the estimations are based on age-specific HPV prevalence (Table 4). On the contrary, in a given population with a larger percentage of cases under age 50, the estimated impact based on age-specific prevalence would be higher than impact based on global estimates if applying the observed age-specific prevalence in our study [6]. A large percentage of cases under age 50 could be seen in high-risk and low-risk populations, in this latter case due to the high percentage of ADC as a result of effective cervical cancer screening.

Age-specific prevalence reveals an important role for HPV types other than HPV 16/18. The higher percentage of HPV-45 in young women (Figure 2) has been previously described in worldwide analyses [17]. As a result, a vaccine including HPV-45 would have greater effect on populations with a higher percentage of under age 50 cases (Table 4), but, due to the higher participation of HR-HPV types other than 16/18 among high-risk populations, this effect would be more likely to occur in developing countries (especially Latin America) than in developed ones. 

In summary, we obtained similar results as those found in previous Latin American surveys, but we observed two relevant factors with the potential to influence the impact of HPV vaccines. Our data findings on multiple infections and age-specific prevalence are consistent with the scarce data on these topics and have further brought to light the role of HPVs other than 16/18. If the impacts of the HPV-18 vaccination were to diminish due to replacement in multiple infections, cross-protection for HPV-45 would be an asset [27]. In addition, a protective effect against HPV-45 would ensure greater impact of current vaccines given the age-specific prevalence (up to 14% percent gained over global estimates for HPV 16/18 with an age-specific incidence as in Connecticut—Table 4).

Although if no cross-protection against HPV-45 could be fully demonstrated, our data highlight the necessity for well-designed future generation vaccines. Immune interference when adding HPV types might have negative effects on vaccine efficacy or duration of protection [27, 28]. Therefore, if a global vaccine with over 90% protection were not possible, a good definition of target populations based on HPV epidemiology would be required to obtain the greatest impact with the lowest number of HPV types (developed and developing countries, young and middle-age women, regions of the world, etc.).

Our results challenge the discussion on HPV vaccine impact in developing nations where factors such as lower vaccine coverage and screening compliance will decrease population effect of vaccination. Hence, we would encourage the use of our data for assessing vaccine cost-effectiveness in Colombia and other Latin American countries. Yet, reports on multiple infections must attain a more standardized structure in future studies and findings on single LR-HPV infections need to be better understood, where differences between phylogenetic and epidemiologic classifications may play a part.

##  Novelty 

This is the largest study on the topic in Colombia and Latin America. With an excellent PCR technique sensitivity for HPV detection, key issues previously narrowly analyzed such as multiple infections, age-specific prevalence, and the role of HPVs other than 16/18, are demonstrated as relevant factors influencing HPV vaccine impact among high-risk cervical cancer populations (developing countries). The data are useful to improve cost-effectiveness analyses and to discuss second generation HPV vaccines.

## Figures and Tables

**Table 1:** Main variables in study population.

Characteristics	Bogota	Barranquilla	All cases
	(*n* = 123)	(*n* = 94)	(*n* = 217)
Average age (SD)	50.9 (14)	52.3 (13.9)	51.5(14)
Histologic type			
Squamous cell carcinoma (SCC)	107 (87%)	78 (83%)	185 (86%)
Adenocarcinoma (ACC)	12 (10%)	11(12%)	23 (11 %)
Unspecified	4 (3%)	5 (5%)	8 (4%)
Distribution of SCC			
Keratinizing (Well-differentiated)	31 (29%)	10 (13%)	41 (22%)
Non-keratinizing	76 (71%)	68 (87%)	144 (78%)
Distribution of ACC			
Endocervical	12 (100%)	10 (91%)	22 (96%)
Endometrioid	0 (0%)	1 (9%)	1 (4%)

Histologic classification based on 14.

**Table:** (a) HPV type-specific prevalence

HPV type	Bogota			Barranquilla		
	Single	All	95%CI	Single	All	95%CI
High risk						
Any HR-HPV	81.3	100.0	—	85.1	98.9	97.2–100
HPV-16	48.8	50.4	42.2–58.6	45.7	52.1	43.6–60.6
HPV-18	4.9	13.8	8.1–19.5	7.4	9.6	4.5–14.6
HPV-45	7.3	9.7	4.9–14.6	6.4	6.4	2.2–10.5
HPV-31	5.7	7.3	3.0–11.6	3.2	4.2	0.8–7.7
HPV-58	5.7	6.5	2.4–10.6	3.2	4.2	0.8–7.7
HPV-33	1.6	4.0	0.8–7.3	4.3	5.3	1.5–9.1
HPV-56	1.6	3.2	0.3–6.2	1.0	3.2	0.2–6.2
HPV-59	1.6	2.4	0.0–5.0	3.2	3.2	0.2–6.2
HPV-51	0.8	1.6	0.0–3.7	2.1	3.2	0.2–6.2
HPV-52	—	0.8	0.0–2.3	2.1	4.2	0.8–7.7
HPV-35	0.8	0.8	0.0–2.3	3.2	3.2	0.2–6.2
HPV-68	1.6	1.6	0.0–3.7	—	1.0	0.0–2.8
HPV-39	—	1.6	0.0–3.7	1.0	1.0	0.0–2.8
HPV-82	—	—	—	1.0	1.0	0.0–2.8
HPV-73	—	0.8	0.0–2.3	—	—	—
Low risk						
Any LR-HPV	—	4.9	1.3–8.4%	1.0	3.2	0.2–6.2
HPV-44	—	3.2	0.3–6.2	1.0	1.0	0.0–2.8
HPV-42	—	0.8	0.0–2.3	—	2.2	0.0–4.5
HPV-72	—	0.8	0.0–2.3	—	—	—
HPV-61	—	0.8	0.0–2.3	—	—	—
Probable high risk						
HPV-26	—	5.7	1.9–9.5	—	5.3	1.5–9.1
HPV-66	0.8	1.6	0.0–3.7	—	—	—
HPV-53	—	0.8	0.0–2.3	1.0	2.1	0.0–4.5
Undetermined risk*						
HPV-34	—	0.8	0.0–2.3	—	1.0	0.0–2.8
HPV-83	—	0.8	0.0–2.3	—	—	—

**Table:** (b) Prevalence of multiple infections

HPV type	Bogota		Barranquilla	
	%	95%CI	%	95%CI
High risk*I*High risk				
HPVs 16-31	0.8	0.0–2.2	1.1	0.0–2.8
HPVs 16-others	0.8	0.0–2.2	1.1	0.0–2.8
HPVs 18-39	0.8	0.0–2.2	—	—
HPVs 18-45	0.8	0.0–2.2	—	—
HPVs 33-53	—	—	1.1	0.0–2.8
HPVs 45-53	0.8	0.0–2.2	—	—
HPVs 51-56	0.8	0.0–2.2	1.1	0.0–2.8
HPVs 58-68	—	—	1.1	0.0–2.8
HPVs 73-31-33	0.8	0.0–2.2	—	—
High risk/Probably high risk				
HPVs 16-26	—	—	3.2	0.2–6.2
HPVs 18-26	5.7	1.9–9.5	2.1	0.0–4.6
HPVs 52-66	0.8	0.0–2.3	—	—
High risk*I*Undetermined risk*				
HPVs 18-83	0.8	0.0–2.3	—	—
HPVs 33-34	0.8	0.0–2.3	—	—
HPVs 52-34	—	—	1.1	0.0–2.8
High risk*/*Low risk				
HPVs 16-42	—	—	1.1	0.0–2.8
HPVs 18-44	0.8	0.0–2.3	—	—
HPVs 45-42	0.8	0.0–2.3	—	—
HPVs 56-others	0.8	0.0–2.3	1.1	0.0–2.8
HPVs 59-44	0.8	0.0–2.3	—	—
HPVs 33-72	0.8	0.0–2.3	—	—
HPVs 58-44-61	0.8	0.0–2.3	—	—

Classifiaction based on 19. Prevalence as percentage of cases for single infections, all infections (single and multiple), and multiple infections.

*HPV types without proper evaluation in case control studies but classified as low risk according to the phylogenetic origin.

**Table 3:** HPV distribution according to histological characteristics.

HPV type	Squamous cell carcinoma		Adenocarcinoma	
	%	C195%	%	C195%
HPV-16	51.5	43.4–59.6	46.8	25.4–68.3
HPV-18	10.8	3.8–17.7	32.9	10.9–54.9
HPV-45	9.0	4.7–13.3	—	—
HPV-31	4.5	1.2–7.8	14.9	1.1–28.7
HPV-58	6.6	2.7–10.6	—	—
HPV-33	4.5	1.2–7.8	—	—
HPV-56	2.8	0.2–5.7	2.7	1.8–7.2
HPV-59	2.8	0.2–5.7	2.7	1.8–7.2
HPV-52	1.0	0.0–2.0	—	—
HPV-35	1.7	0.1–3.4	—	—
HPV-44	0.3	0.2–0.9	—	—
Other HR	4.5	—	—	—

Estimations are based on 208 cases (SCC 185, ADC 23).

Single infections assumed after distribution of multiple infections.

**Table:** (a) Cervical cancer incident cases

Country	Group of age			Total*
	<50	50–59	≥60	
Colombia, Cali	489	217	339	1045
Uganda, Kyadondo County	313	69	70	452
USA, Connecticut	382	52	46	480

**Table:** (b) Expected incident cases and estimated impact of vaccination

Case	<50	50–59	≥60	Impact based on global estimates	Impact based on age-specific estimates
HPV 16,18					
Attributable cases (%)	65.5	77.1	46.3	63.2	—
Colombia, Cali	169	50	182	63.2	61.7
Uganda, Kyadondo County	108	16	38	63.2	64.3
USA, Connecticut	132	12	25	63.2	64.9
HPV 16,18,45					
Attributable cases (%)	79.8	79.4	48.6	71.4	—
Colombia, Cali	99	45	174	71.4	69.6
Uganda, Kyadondo County	63	14	36	71.4	74.9
USA, Connecticut	77	11	24	71.4	76.8
HPV 16,18,45,31					
Attributable cases (%)	85.2	80.6	58.4	76.8	—
Colombia, Cali	72	42	141	76.8	75.6
Uganda, Kyadondo County	46	13	29	76.8	80.3
USA, Connecticut	57	10	19	76.8	82.1
HPV 16,18,45,58					
Attributable cases (%)	81.6	87.4	60.6	77.3	—
Colombia, Cali	90	27	134	77.3	76.0
Uganda, Kyadondo County	58	9	28	77.3	79.2
USA, Connecticut	70	7	18	77.3	80.2

Incident cases in (a) based on 28. Attributable cases based on study results. Numbers for each country in (b) correspond to the expected number of cases given the percentage of attributable cases. The impact based on global estimates was obtained from cumulative percentages in Figure 1(a). The impact based on age-specific estimates corresponds to the reduction in number of cases as percentage of the initial number of cases, obtained from the summatory of expected cases in age groups (<50, 50–59, ≥60). Single infections assumed after distribution of multiple infections.

*Total cases do not include unknown age.
